# Further evaluation of the Walter Reed Visual Assessment Scale: correlation with curve pattern and radiological deformity

**DOI:** 10.1186/1748-7161-2-12

**Published:** 2007-09-23

**Authors:** Juan Bago, Jose M Climent, Sonia Pineda, Carmen Gilperez

**Affiliations:** 1 Spine Unit. Hospital Vall d'Hebron, Barcelona, Spain; 2 Department of Physical Medicine and Rehabilitation, Hospital Universitario. Alicante, Spain; 3 Department of Physical Medicine and Rehabilitation, Hospital Vall d'Hebron, Barcelona, Spain

## Abstract

**Background:**

The Walter Reed Visual Assessment Scale (WRVAS) was designed to measure physical deformity as perceived by patients with idiopathic scoliosis. Previous studies have shown that the instrument has excellent internal consistency and a high correlation with the radiological magnitude of scoliotic curves. Nonetheless, it is not known whether the scale can discriminate between the various curve patterns of the deformity, or whether the deformities represented in the scale's drawings relate to the corresponding radiological deformities.

**Methods:**

This study included 101 patients (86 women and 15 men; mean age 19.4 years) with idiopathic scoliosis. In a single visit, patients underwent standing PA radiography of the spine and completed the WRVAS. X-ray measurements included: 1) magnitude (Cobb angle) of the proximal thoracic curve (PT), main thoracic curve (MT), and thoracolumbar/lumbar curve (TL/L); 2) difference in shoulder level; 3) T1 offset from the central sacral line (T1-CSL); 4) apical vertebra (apV) rotation at the MT and TL/L curves and 5) apical vertebra offset of the MT and TL/L curves from the central sacral line. A variable designated *Cobbmax *was defined as the largest angle of the three curves (PT, MT or TL/L). Patients were grouped onto three patterns: Thoracic (TH Group)(n = 30, mean MT 42.1°, TL/L 20.9°); double major (DM Group) (n = 39, mean MT 38.6°, TL/L 34.4°) and thoracolumbar (TL Group)(n = 32, mean MT 14.3°, TL/L 25.5°). The magnitude of the curves in the TL Group was significantly smaller than in the other groups (*P *< 0.05). The Spearman partial correlation coefficient was determined between the score for each WRVAS question and the curve pattern, adjusting for the *Cobbmax *variable. The Spearman correlation coefficient was determined between the WRVAS items and shoulder imbalance, T1-CSL offset, MT Cobb angle, MT apV rotation, MT apV offset, PT Cobb, TL/L Cobb, TL/L apV rotation and TL/L apV offset.

**Results:**

The median (interquartile range) of the total WRVAS score was 14 (IQR 6). No correlation was found between the curve pattern and the various scores on the scale (partial correlation coefficients ranged from -0.16 to 0.12). WRVAS drawings for items 1, 2, 4 and 7 correlated satisfactorily with the corresponding radiological measurements (correlation coefficients, 0.62, 0.3, 0.48 and 0.53, respectively). Items 3, 5 and 6 did not correlate with the radiological measurements (correlation coefficients -0.06, -0.07 and 0.05, respectively).

**Conclusion:**

The profile of the individual WRVAS scores does not differentiate among specific curve patterns (thoracic, double major and thoracolumbar/lumbar). Moreover, some of the drawings (items 3, 5 and 6) do not correlate with the radiological deformity they were designed to measure.

## Background

**Figure 1:**
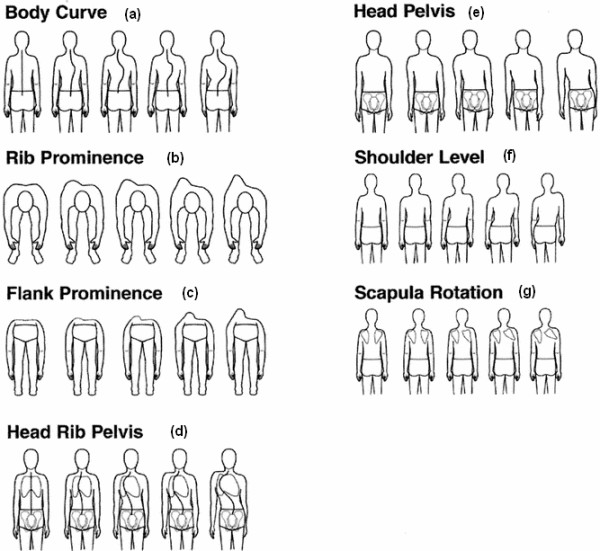
The Walter Reed Visual Assessment Scale (used with permission from Sanders et al [1]).

The Walter Reed Visual Assessment Scale (WRVAS) (Fig. 1) was designed to measure physical deformity as perceived by patients with idiopathic scoliosis. The scale assesses seven aspects of the deformity: spinal curvature, rib prominence, flank prominence, deformity/alignment of the thorax with respect to the pelvis, trunk imbalance, shoulder asymmetry and scapular asymmetry. In the initial publication, it was demonstrated that the WRVAS scores correlated with the magnitude of the curve and were clearly different in relation to curves less than and greater than 30 degrees. Moreover, the instrument differentiated between patients stating that they "noticed" the deformity from those stating that they "did not notice" it [1].

Following that publication, our group performed an in-depth evaluation of the metric characteristics of the scale [2]. The internal consistency was found to be excellent, with no differences observed between patients more or less than 18 years of age. Analysis of the distribution of the scores showed a somewhat elevated floor effect in some of the questions, a fact indicating that perception of the deformity is inconsequential for small curves. In keeping with the findings of Sanders et al [1] the correlation between the WRVAS scores and the radiological magnitude of the curves was high. The analysis of convergent validity demonstrated a significant correlation between the WRVAS questions and the self-image scale in the SRS-22 questionnaire. Along this line, the correlation between the WRVAS and the SRS-22 pain, function and mental health scales was only marginal, an indication that the WRVAS is a specific scale for assessing physical aspects if the deformity in patients with idiopathic scoliosis.

At completion of this analysis, we formulated a series of questions related to the practical utility of the scale. First, it seemed interesting to determine whether the test would be able to discriminate between the various scoliotic curve patterns. The hypothesis formulated was that patients with different curve patterns (thoracic vs. lumbar. vs. double curves) should have different scores for some of the items on the scale. Second, we wanted to determine the relationship between the various figures comprising the scale and the corresponding radiological deformities. Thus, the aims of the present study were to assess the impact of curve pattern on the WRVAS scores and establish the relationships between the scores for the various questions and the corresponding radiological measurements.

## Methods

This a cross-sectional, observational study, approved by the Medical Ethics Committees of the participating hospitals. The study included patients with idiopathic scoliosis, 10 to 40 years of age, consecutively enrolled in two centers. Patients who had undergone surgical treatment were excluded. The sample included 101 patients (86 women and 15 men) with a mean age of 19.4 years (range 12–40 years). After giving informed consent for participation, all patients completed the Walter Reed Visual Assessment Scale [1] (Fig. 1). This instrument includes a group of drawings representing seven aspects of the scoliotic deformity: item 1 (WR1), spinal deformity (Fig. 1a); item 2 (WR2), rib prominence (Fig. 1b); item 3 (WR3), flank prominence (Fig. 1c); item 4 (WR4), thoracic deformity (Fig. 1d); item 5 (WR5), trunk imbalance (Fig. 1e); item 6 (WR6), shoulder asymmetry (Fig. 1f); and item 7 (WR7), scapular asymmetry (Fig. 1g). Each aspect of the deformity is shown with five levels of increasing severity that are scored from a minimum of 1 to a maximum of 5. Results are presented as the sum of the seven questions (Wr total). During the same visit, a standing PA radiograph of the spine was obtained for each patient, which was used to carry out the following measurements: 1) magnitude (Cobb angle) of the proximal thoracic curve (PT), main thoracic curve (MT), and thoracolumbar/lumbar curve (TL/L); 2) T1 tilt; 3) difference in shoulder level (a line perpendicular to the central sacral line was drawn from the point where the clavicle crossed the chest cage, and the difference in height between the right and left was recorded) [3]; 4) T1 offset from the central sacral line (T1-CSL); 5) apical vertebra (apV) rotation at the MT and TL/L curves, as determined by the trigonometric method of Stokes et al [4]; and 6) apV offset of the MT and TL/L curves from the central sacral line [5]. The right-hand axis convention (right-hand rule) was used to determine the signs of these angles in the frontal and transversal planes [6]. A variable designated *Cobbmax *was defined as the largest angle of the three curves (PT, MT and TL/L).

### Patient grouping

Based on the radiological data, the type of curve was classified according to Lenke's classification [7]: 25 were Type 1, 8 Type 2, 35 Type 3, 4 Type 4, 11 type 5, and 21 Type 6. Departing from this basis, three groups were established: the first included Lenke types 1 and 2 and was labeled thoracic pattern (Th Group, n = 30); the second included patients with a double major curve (DM Group), classified as Lenke 3 and 4 (n = 39), and the third had a thoracolumbar pattern (TL Group) and consisted of patients with curves classified as Lenke 5 and 6 (n = 32).

To assure that patient grouping was accurate, the ratio of the MT curve to the TL/L curve (Th/TL ratio) was determined. The hypothesis was that if the patient grouping were correct, the Th/TL ratio should be close to 1 for the DM pattern, greater than 1 for the TH pattern and less than 1 for the TL pattern. Mean Cobb angle was 31.9° for the MT curve, 27.6° for the TL/L curve and 36.1° for the Cobbmax. The Th/TL ratio was 2.00 for the TH group, 1.08 for the DM group and 0.54 for the TL group (ANOVA, F = 207, *P *= 0.0001), thereby confirming that patient grouping was accurate. The mean magnitude of the MT and TL/L curves, and the Th/TL ratio for each pattern are shown in Table 1.

**Table 1:** Mean magnitude (± standard deviation) of the main thoracic curve and thoracolumbar/lumbar curve and the Th/TL ratio for each of the three curve patterns

	TH pattern	DM pattern	TL pattern
MT Curve	42.1 (± 17.2)	38.6 (± 20.3)	14.3 (± 8.4)*
TL/L Curve	20.9 (± 7.9)	34.4 (± 12.6)*	25.5 (± 10.3)
Cobbmax	42.1 (± 17.2)	40.1(± 19.5)	25.5 (± 10.3)*
Th/TL ratio	2.0 (± 0.39)*	1.08 (± 0.24)*	0.54 (± 0.19)*

*Mean significantly different as compared to the other groups (*P *< 0.05)

### Statistical Analysis

#### Impact of curve pattern on the WRVAS

The median of the three groups were compared with Westenberg-Mood median test. To assess the relationship between the curve pattern and WRVAS scores, eliminating the influence of curve magnitude, we determined the Spearman partial correlation coefficient between the score for each WRVAS question and the variable *Th/TL ratio *(as an indicator of the curve pattern), controlling for the *Cobbmax *variable.

#### Relationship between the WRVAS and radiological variables

The non-parametric Spearman correlation coefficient was determined between the WRVAS items and the following radiological variables: shoulder imbalance. T1-CSL offset, MT Cobb angle, MT apV rotation, MT apV offset, PT Cobb, TL/L Cobb, TL/L apV rotation and TL/L apV offset. Significance was determined with Student-t test. Data were analyzed using SPSS for Windows, version 11.5. To determine the Spearman partial correlation coefficient SAS Program was used. Significance was set at < 0.05.

## Results

The mean and range of the radiological measurements in the frontal plane are summarized in Table 2. The median of the total WRVAS score was 14 (interquartile range IQR 6). The median (and IQR) for each of the seven questions were: item ***1***, 3 (1); item ***2***, 2 (1); item ***3***, 2(0); item ***4***, 2(1); item ***5***, 2(1); item ***6***, 2(1) and item ***7***, 2(1).

**Table 2:** Means and range of values obtained for the radiological measurements in the frontal plane

T1-CSL (mm)	0.02	-53 – 50
Shoulder balance (mm)	-0.7	-22 – 20
Proximal thoracic Cobb (°)	18.6	3 – 53
Main thoracic Cobb (°)	31.9	0 – 98
Thoracolumbar/Lumbar Cobb (°)	27.6	8 – 60
Cobbmax (°)	36.1	10 – 98
MT apical vertebra offset (mm)	-19.5	-102 – 19
MT apical vertebra rotation (°)	6.9	-14 – 40.2
T1 tilt (°)	1.2	-12 – 17
TL/L apical vertebra offset (mm)	11.5	-46 – 53
TL/L apical vertebra rotation (°)	-5.1	-34 – 16

### Impact of the curve pattern

The median for each WRVAS question and the sum of all the scores are shown in Table 3. The TL group showed significantly lower values for questions 2, 3, 6, 7 and the total score. This result, however, coincides with the finding that the Cobbmax in this group was significantly lower then in the others (ANOVA, *P *< 0.05)(Table 1). Because of the influence of the Cobbmax on the scores for the various questions (Table 4), it was necessary to adjust for the effect of curve magnitude when analyzing the impact of curve pattern on WRVAS scores. To this purpose, we determined the Spearman partial correlation coefficient between the score for each WRVAS question and the variable Th/TL ratio (as the indicator of curve pattern) controlling for the Cobbmax. The partial Spearman correlation coefficients obtained were not significant for any of the seven questions or for the total score (partial *rho *value -0.16 to 0.12). This indicates that the various scores in the TL group were influenced by the magnitude of the curve and that the impact of the curve pattern was null.

**Table 3:** Medians of WRVAS questions for each curve pattern

	WR1 Spinal deformity	WR2 Rib promin	WR3 Flank promin	WR4 Thoracic deformity	WR5 Trunk imbalance	WR6 Shouolder asymmet	WR7 Scapular asymmtr	WR Total
Group TH	3.0	2.0	2.0	2.0	2.0	2.0	2.0	15.5
Group DM	3.0	2.0	2.0	2.0	2.0	2.0	2.0	16.0
Group TL	2.0	1.0*	2.0*	1.0	2.0	2.0*	2.0*	13.0*

*Groups significantly different (*P *< 0.05)

**Table 4:** Correlation coefficient (Spearman) matrix among WRVAS items and radiologic measurements

	WR1 Spinal deformity	WR2 Rib promin	WR3 Flank promin	WR4 Thoracic deformity	WR5 Trunk imbalance	WR6 Shouolder asymmet	WR7 Scapular asymmtr	Walter Reed Total
T1-CSL	-.04	-.15	-.14	-.03	-.07	-.17	-.12	-.11
Shoulder imbalance	-.01	-.13	-.08	.01	-.14	.05	-.14	-.09
PT Cobb curve	.49**	.39**	.27*	.40**	.29**	.23*	.38**	.44**
MT Cobb curve	.62**	.53**	.42**	.48**	.41**	.40**	.53**	.61**
MT apV offset	-.47**	-.47**	-.39**	-.31**	-.29**	-.40**	-.44**	-.50**
MT apV rotation	.33**	.30**	.19	.15	.37**	.21	.33**	.33**
TL/L Cobb curve	.60**	.37**	.36**	.31**	.42**	.25*	.41**	.51**
TL/L apV offset	.18	-.07	.07	.00	.10	-.10	.02	.05
TL/L apV rotation	-.15	.00	-.06	-.11	-.16	-.09	-.09	-.15
Cobbmax	0.68**	0.49**	0.40**	0.48**	0.46**	0.37**	0.51**	0.62**

*P < 0.05

**P < 0.01

### Relation between radiologic parameters and WRVAS scores

Spearman correlation coefficients between each WRVAS question and the radiologic measurements are shown in Table 4. **Item 1**. This question refers to the spine deformity. A strong correlation was found between MT Cobb (rho = 0.62), TL/L Cobb (rho = 0.60) and PT Cobb (rho = 0.49). **Item 2**. Refers to the magnitude of the rib prominence. This item correlated with MT Cobb (rho = 0.53), and to a lesser degree with MT apV offset, (rho = -0.47) and MT apV rotation (rho = 0.30). **Item 3**. Assesses magnitude of the flank prominence. A moderate correlation was found with MT Cobb (rho = 0.42) and TL/L Cobb (rho = 0.36); in contrast, no significant correlation was found with TL/L apV rotation or offset. **Item 4**. Refers to deformity/asymmetry of the rib cage. This item correlated satisfactorily with the MT curve variables (Cobb rho = 0.48; apV offset rho = -0.31). **Item 5**. Refers to head-pelvis alignment; hence the radiological variable to assess this aspect would be T1 offset from the central sacral line. This item correlated with the magnitude of MT Cobb (rho = 0.41) and TL/L Cobb (rho = 0.42) curve variables, but there was no significant correlation with T1-CSL offset (rho = -0.07). **Item 6**. Assesses shoulder level imbalance. A moderate correlation was found with magnitude of the MT curve (rho = 0.4), but there was no correlation with the shoulder level imbalance (rho = 0.05). **Item 7**. Refers to scapular asymmetry. A significant correlation was found with the components of the MT curve (Cobb rho = 0.53, apV offset rho = -0.44 and apV rotation rho = 0.33). **The sum of all the scores **correlated with the magnitude of the PT curve (rho = 0.44), MT curve (rho = 0.61), and TL/L curve (rho = 0.51) and the variable Cobbmax (rho = 0.62).

## Discussion

The WRVAS is a valid test for recording the subjective perception scoliosis patients have of their deformity [1,2]. Nevertheless, according to the results of the present study, the profile of scores obtained with the WRVAS does not allow differentiation among the various curve patterns occurring in this condition. What is more, some of the deformities represented in the figures comprising the instrument do not correspond with the radiological measurements of the deformity depicted. These facts generate some doubt as to the full validity of the scale.

### Limitations of the study

Stratification of the patient sample in this study was based on the curve pattern. Patients were categorized according to the classification of Lenke [7] because this system allows classification of the scoliotic curves in broad terms into thoracic (types 1 y 2), double curves (types 3 and 4) and thoracolumbar/lumbar (types 5 and 6). This method of grouping the patients may be debatable. It could be argued that it would have been preferable to have enough cases to represent all six types of curves. This would have considerably lengthened patient enrollment for the study, however, since the prevalence of some curve patterns (e.g., type 4) is quite low. Moreover, in our opinion, it is difficult for a patient to perceive the visual difference between, for example, Lenke types 5 and 6. The grouping applied seems to have been effective since the relationship between the magnitudes of the thoracic and thoracolumbar/lumbar curves between the three groups was dissimilar. Nevertheless, stratification by curve pattern led to an undesired effect: the magnitude of the main curves was different between the groups, specifically the mean magnitude of the curve in the group with the thoracolumbar/lumbar pattern was significantly smaller than that of the other groups.

### Relationship between the WRVAS and curve pattern

Our results show that the WRVAS cannot discriminate between the various curve types. The crucial variable that determines the score on the WRVAS is the magnitude of the curve and not the pattern of the curve (thoracic, thoracolumbar or double major). As is seen on Table 3, the profiles of the scores among the groups are virtually identical. If the scale were able to discriminate, it would be expected that the profiles would be different. For example, in the TL group, we would expect that the score for item 3 (flank prominence) would be higher than the score for item 2 (rib prominence), whereas in the TH group the opposite should occur; or, we might expect that in the TL group, item 3 would be clearly higher than item 7 (scapular asymmetry). Our data contradict the impression voiced by some experts that the aesthetic impact of double curves would be less than that of single curves.

### Relationship between the WRVAS figures and the radiological variables

One problem we faced when designing the study was to establish a gold standard pattern to be used for determining the validity of each of the seven figures. Classically, spine deformity is assessed by clinical examination and radiological measurements. The textbooks usually recommend that data on shoulder, scapula and waist asymmetries, trunk imbalance, and the angle of trunk inclination be determined from the clinical examination [8,9]. Nevertheless, the maneuvers for performing the examination are not well-standardized and, in general, their reliability is uncertain. The only such maneuver that seems to have acceptable reliability is measurement of the angle of trunk inclination with a scoliosis inclinometer (scoliometer)[10,11]. The reliability of C7-plumbline deviation has been assessed [12] and seems to be less dependable than the scoliometer measurement. We were not able to gather information on the reliability of other examinations, such as the difference in shoulder height or waist crease. The position of the scapulas can be reliably measured with the Lennie test [13]. However, this has only been tested in individuals without spine deformity.

The radiological measurements seem to have undergone a more thorough evaluation. Recent studies have shown that most of the parameters usually determined on PA radiographs of the spine (Cobb angle, apical vertebral offset) have excellent interobserver and intraobserver reliability [5,14,15]. Apical vertebra rotation was measured in the present study with a trigonomic method that has shown good precision [4], whereas differences in shoulder level were determined with an adequately reliable method [3]. Thus, we opted to correlate the WRVAS measurements with several radiological parameters that describe the deformity because they seemed more reliable than clinical assessment.

Questions 1 and 2 correlated satisfactorily with the corresponding radiological variables. Question 3, which refers to flank prominence, should have correlated with the magnitude and apV rotation of the lumbar curve. We found, however, that Question 3 related with the main thoracic curve and that the correlation was weak with lumbar curve magnitude and non-existent with lumbar apV rotation. Hence, we are led to consider that Question 3 does not assess the deformity it is designed to assess.

The deformity that Question 4 attempts to evaluate is somewhat uncertain. In the original description [1], the question is labeled "Head Rib Pelvis". Attending to the figures, this item seems to refer to the alignment between the head, rib cage and pelvis. However, the lungs are also drawn in, giving the impression that the figure attempts to evaluate the rib cage deformity. According to the orientation of the scoliosis, it seems to be a frontal view, although the patient's face is not depicted. Thus, we are led to consider that Question 4 assesses the deformity of the thoracic area. Nevertheless, we have the impression that the face validity of this question is debatable.

Question 5 focuses on trunk imbalance and should relate with offset of T1 to the central sacral line. Nevertheless, the correlation between these two variables was not significant, casting doubts on the validity of the question. Question 6 centers on shoulder imbalance and, logically, should relate with its radiological counterpart. However, once again, there was no correlation between the score and the radiological measurements. Lastly, Question 7 refers to scapular asymmetry, for which a radiological equivalent has not been determined. In bivariate analysis, the scores for this item correlated significantly with the magnitude of the MT curve; thus, it can be considered a good estimation of thoracic deformity.

To summarize, items 1, 2, 4 and 7 of the WRVAS showed a satisfactory relationship with the deformity of the thoracic area, which is what they were designed to measure. Items 3 and 6 exhibited a clear absence of correlation with the deformity they should be measuring and can be considered to have questionable validity. In fact, these items showed the weakest correlation with the Cobbmax variable (Table 4). Question 5 did not relate with its radiological counterpart (T1-CSL offset) but showed a good correlation with the Cobbmax. We have the impression that patients relate this question with waist asymmetry, an aspect that is not specifically covered by any of the figures provided.

The results of this study indicate that the WRVAS is lacking in some aspects. First, it seems clear that the scale mainly assesses the deformity of the thoracic area, whereas the lumbar deformity (both flank prominence and waist asymmetry) are poorly represented. Second, the WRVAS represents the various deformities in only one direction. For, example shoulder imbalance is depicted as a range from normal to maximum elevation of the right shoulder. The possibility that the left shoulder might be elevated is not contemplated. This fact would undoubtedly explain the lack of correlation between the radiological measurement and the score for item 6. This problem is repeated for virtually all the questions. The solution is difficult because it would require the use of different questionnairs according to the direction of the deformity or the requirement that each item range from the maximum left deformity to the maximum right deformity. This might very well compromise the practical utility of the scale. Finally, the scores for various questions do not seem to correspond to what the patient "sees in the mirror". Rather, they seem to correspond more to the subjective impression patients have of their back (which they usually do not see), and this impression is mainly based on the spinal x-rays.

Most authors agree that it is necessary to record the body image disturbance caused by scoliosis [16,17]. Hence, the efforts to improve the available instruments for this purpose should continue. The SRS-22 body image scale is valid, but shows a weak correlation with the magnitude of the curve. The WRVAS is an improvement in this regard. Based on the known data for the scale (internal consistency, discriminant validity), it seems appropriate to use it for overall assessment of the subjective perception patients have of their deformity. Nevertheless, the validity of the instrument to describe a patient's deformity is clearly insufficient. One potential focus of future work might be to modify some of the WRVAS items that are less valid in this regard. We advocate changes that will yield information on the frontal vision of the body and improve the representation of waist asymmetry. On the other hand, if the total sum of the scale is considered sufficiently valid, it might be worthwhile investigating whether some questions that do not seem to provide valid information (such as items 3 and 6) might be excluded.

## Conclusion

The WRVAS is a valid questionnaire for assessing the subjective perception patients have of their deformity. Nonetheless, the profile of the individual scores did not differentiate among the various curve patterns studied (thoracic, double major and thoracolumbar/lumbar). Moreover, some of the scale's figures (items 3, 5 and 6) did not correlate with the radiological deformity they were designed to measure. These findings indicate that the WRVAS is not valid to describe the actual deformity a patient has.

## Competing interests

The author(s) declare that they have no competing interests.

## Authors' contributions

JB contributed in analysis, interpretation of data and drafting the manuscript

JMC contributed in analysis, interpretation of data and drafting the manuscript.

SP contributed in acquisition of data

CG contributed in revising the manuscript

All authors have read and approved the final manuscript
